# The Status of Marine Mussel Pollution Research in South Africa (2012–2022)

**DOI:** 10.3390/foods12213983

**Published:** 2023-10-31

**Authors:** Deborah Caitlin Firth, Lutz Auerswald, Philip E. Strydom, Louwrens Christiaan Hoffman

**Affiliations:** 1 Department of Animal Science, Stellenbosch University, Cape Town 7600, South Africa; dcfirth21@gmail.com (D.C.F.); lutz.auerswald@gmail.com (L.A.); pestrydom@sun.ac.za (P.E.S.); 2 Department of Forestry, Fisheries and the Environment, Cape Town 8012, South Africa; 3 Centre for Nutrition and Food Sciences, University of Queensland, Brisbane, QLD 4072, Australia

**Keywords:** mussels, pollution, trace elements, organic pollutants, microplastics, South Africa

## Abstract

The growing human population requires more food each year, and seafood products can help meet this demand if clean water resources are available for their growth. Farmed and wild mussels are environmentally friendly seafood with many health benefits to human consumers, but they can also pose a health risk if they are harvested from areas where marine anthropogenic pollution is uncontrolled or unmonitored. While the coastline in South Africa has long been assumed to be pristine, a growing number of recent studies are raising contamination concerns. Baseline studies establish a wide range of anthropogenic pollutants to be present in the marine environment, specifically in urbanised or industrialised areas like major cities or harbours. This review summarises how mussels could pose health risks to human consumers and the current research that is being conducted by private researchers and institutions in South Africa. The review emphasises the need for more research in the field and for governmental pollution monitoring data to be released to the public.

## 1. Introduction

A large percentage of the world’s human population lives within 100 km of a coastline due to the abundance of resources, ease of over-water trade and transport, and the availability of activities of recreational or cultural significance [1]. Estimates have shown that coasts experience almost three times the global average human population density [2], and numerous studies have linked growing human population densities to increasing marine pollution levels and decreasing health of nearby marine ecosystems and organisms [3,4,5,6]. This, in turn, threatens the health and safety of coastal-caught or farmed fish and other traditionally healthy marine resources upon which many humans rely [7], creating a potential food security issue if anthropogenic pollution continues unchecked.

### 1.1. Marine Pollution Increases Human Health Concerns for Seafood Consumers

While current data are scarce, a study on human impacts in the coastal zone noted that in the early 2000s, South Africa already had more than 67 pipelines discharging 300 million m^3^ of wastewater per day into the coastal ocean, with the number of pipes in the surf zone increasing and limited monitoring making trends in ecosystem health hard to discern [8,9]. The first South African Environment Outlook (SAEO) Report in 2006 [8] stated that the oceans and coastal environments of the country were in a moderately healthy state (when compared to international trends), while the second SAEO Report in 2012 classified these same environments as only marginally healthy, a noteworthy change in only 5 years, which is largely attributed to increased land-based pollution [8,10]. The new Green Drop Report from the Department of Water and Sanitation revealed that in 2021, 39% of municipal wastewater systems in the country were within a critical state, a worrying increase from the 29% identified in the previous Report from 2013 [11]. Human wastewater is often released directly into rivers and oceans, and therefore, the worsening state of wastewater treatment works is cause for serious concern to marine and freshwater aquaculture farmers alike. 

Despite the long-held perception that the South African coastline remains relatively pristine and unpolluted by international standards [12], recent biomonitoring studies show that there are potential pollution issues within heavily industrialised areas such as harbours [13,14,15,16], with numerous researchers noting there are insufficient data to make long-term conclusions on whether coastal pollution is improving or worsening over time [15,17,18,19]. Considering the above statements regarding rising human population densities in the coastal zone, combined with the evident decreasing state of effectiveness of South African wastewater treatment plants and their tendency to release untreated effluent into rivers and oceans, unmonitored anthropogenic pollution is cause for significant concern along the coastline, particularly to seafood producers and consumers.

Unpolluted marine waters are essential to all seafood consumers; coastal aquaculture farms and wild harvesters (recreational or subsistence) need healthy ecosystems to ensure healthy seafood products. Marine aquaculture in South Africa occurs either in coastal areas (mussel, oyster, and fish farms) or relies on coastal seawater pumped into land-based facilities (abalone farms) and is thereby heavily reliant on clean seawater for the production of seafood that is safe for human consumption. The wild harvesting of mussels (*Mytilus galloprovincialis*, *Choromytilus meridionalis*, *Perna perna*), white mussels (*Donax serra*), and oysters (*Striostrea margaritacea*) is also popular within many coastal communities in South Africa [20], and permits are easily attainable. Without the correct level of coastal pollution monitoring and intervention, both industries could soon face serious food safety issues that may even result in problems for the entire marine food chain. 

### 1.2. The State of Coastal and Marine Pollution Research in South Africa

O’Donoghue and Marshall (2003) conducted a comprehensive review of marine pollution research in South Africa between 1960 and 2003, with their findings indicating a concerning decrease in published research in the country since it peaked in the 1980s [17]. In the time between 1980 and 1984, there were over 70 published papers on marine pollution research in South Africa, while between 1995 and 1999, less than 20 studies were recorded [17]. A more recent review by Wepener and Degger (2012) found a similar trend, with marine pollution research peaking between the years 1980 and 1989 at almost 150 published studies [18]. There were less than 50 published studies in both of the following time intervals, namely 1990–1999 and 2000–2012 [18]. The potential dwindling of published research on the health of marine coastal ecosystems could result in the underestimation of pollution threats and is a concern for the public, private researchers, and governmental institutions alike. 

### 1.3. Mussels as Biomonitors of Marine Pollution

When monitoring marine coastal pollution, there are three environmental compartments that can be analysed by researchers: the water column, beach or benthic sediments, and marine biota. While water and sediment samples can be simple to collect, they are known to have high variability (due to tides, currents, floods, etc.) and often do not give an indication of the true environmental pollution burden experienced by marine species and potentially the humans who consume them [13,21]. When Goldberg (1975) realised the complexity of monitoring marine anthropogenic pollutant inputs and impacts and the cost this would place on marine scientists, he instead suggested using *Mytilus edulis* mussels (and other similar native or invasive species) as an indicator species for ocean pollution monitoring programs. Since the suggestion of “The Mussel Watch”, mussels have been used as marine pollution monitoring tools for trace elements (TEs), persistent organic pollutants (POPs), and microplastics (MPs) on coastlines worldwide, allowing for global comparisons of contamination levels [22,23,24,25]. While South African mussel contamination levels are included in some international research regarding metal pollution [22], the lack of MP quantification research within local biota means that neither Africa nor South Africa was included in a recent global MP review [23], indicating that South Africa may not be keeping up with international trends in this regard.

#### 1.3.1. The Benefits of Mussels as Coastal Biomonitors

There are multiple reasons why mussels make excellent biomonitors; the first is that mussels are ubiquitous, and commercially important mussel species are found on all continents [24,26]. This allows for studies conducted across different continents to be somewhat comparable and further increases the effectiveness and insight of a worldwide monitoring scheme [27]. Their reproductive strategy (broadcast spawning) ensures that mussels form the base of numerous marine food chains worldwide; they are, therefore, often found in large numbers (mussel beds) within predictable locations [26]. This makes repeat sample collection relatively easy and allows for the short- and long-term monitoring of changes in pollutant concentrations within marine environments [24]. Their place at the bottom of the food chain means mussel contamination analyses can help quantify direct pollution threats to human consumers as well as give an idea of the pollution exposure levels of higher trophic level species from the same environment (e.g., lobsters, fish, sharks). 

Mussels are also an easy marine species for researchers to gather, as they are sessile and often form dense beds in the subtidal and intertidal zones, which are accessible during low tide. Researchers do not need boats or special equipment to collect, handle, or sacrifice mussel samples, making their collection cost highly effective (an important factor in encouraging repeat studies). Their sedentary nature means that they are more accurate indicators of local pollution, as opposed to more mobile species like fish [24]. While analysing highly mobile and migratory marine biota (e.g., fish, sharks) can give a clear idea of the direct pollution threat to human consumers, this still creates a problem for pollution monitoring studies because it often cannot be stated with certainty that pollutants detected within the flesh of caught specimens were accumulated at the same point where the sample was collected. This makes it harder to identify where the original pollution occurred, and an essential function of pollution monitoring programs is commonly to identify the point-source location or cause of detected pollution so that it can be addressed by the relevant stakeholders. 

Where mussels truly stand out as an indicator species is their potential to gather and store a wide range of marine pollutants within their flesh. Due to being filter feeders who receive all their nutrition out of oceanic waters, mussels are constantly exposed to any pollutants present and tend to accumulate and concentrate these during normal feeding processes [24,28]. In 1972, mussels had already been found to be accurate sentinels for hydrocarbon pollution because they rapidly take up and store both saturates and aromatics with minimal metabolic breakdown [29]. Mussels also accumulate and store both essential and toxic metals found in the water column, often at levels exceeding those in the ocean itself [30]. Lastly, mussels are known to be hardy and often survive in locations too polluted for more sensitive species, and they can be transplanted into areas where natural populations are not present to further expand potential research areas [24,31,32]. Mussels’ well-documented ability to accumulate and then concentrate toxic metals and POPs in the water column can also result in potential human health risks that may need to be mitigated by consumption limits [3,33,34,35].

#### 1.3.2. The Challenges of Mussels as Coastal Biomonitors

While mussels are currently accepted as one of the best sentinel organisms to monitor marine pollution, they also pose certain challenges to researchers, the first being that even established mussel beds can disappear or shift location according to water temperatures, food availability, or the presence of invasive or predatory species. This could negatively impact researchers’ ability to sample repeatedly in the same area over years or decades, but this limitation can potentially be overcome through the transplanting of mussels or the use of “artificial mussels” [31,36,37]. Prior research has also identified differences in contamination and high inter-individual variability between different mussel species, sexes, seasons, and sizes (discussed below). To increase the comparability of global studies using mussels as bio-monitors, researchers need to consider the causes or mechanisms behind these significant differences and ensure that they are considered and controlled for in future studies.

Species: The mussels found along the South African coastline are known to have different distributions, growth rates, filtration capacities, spawning seasons, reproductive outputs, and lifestyle adaptations [38,39,40,41]; these factors could significantly affect the extent to which certain species are exposed to and accumulate contaminants within the water column and sediment. *Perna perna* and *C. meridionalis* mussels collected from the same locations along the Namibian coastline were found to have accumulated Cd, Cr, and Zn to significantly different concentrations [42]. Mussels from Saldanha Bay (South Africa) aquaculture facilities demonstrated that invasive *M. galloprovincialis* accumulated TEs (Al, Cr, Fe, Zn, Cd, and Pb) and two polychlorinated biphenyl (PCB) congeners (118 and 149) at greater concentrations than those found within the local *C. meridionalis*, which in turn accumulated more Cu and Mn [14,43]. A recent Cape Town study found wild *M. galloprovincialis*, *C. meridionalis,* and *A. ater* mussels collected from the same locations to accumulate MP particles at similar rates [44].

Sex: Mature female *C. meridionalis* had metal concentrations~twice those of mature males [45,46]. Thirteen of nineteen TEs analysed in *M. galloprovincialis* were significantly higher within the flesh of female mussels [47]. While the cause of these sexual differences is yet to be determined, a similar pattern was identified in *Mytilus edulis* mussels, where female mussels had far greater flesh concentrations of Mn, Cd, and Zn [48]. 

Season or Year: Mussels experience significant seasonal fluctuations in tissue composition, with these changes closely linked to their sexual cycle and gametogenesis, as well as the availability of food [28,49,50]. Some contaminant studies have even suggested that trace element content does not change seasonally but rather that the massive build-up of gametes prior to spawning causes an overall dilution of TEs within mussels’ flesh [47,51]. A review of Cape Town Mussel Watch Programme data (collected between 1985 and 2008) on *M. galloprovincialis* showed Cd, Cu, Fe, Pb, Mn, and Hg concentrations fluctuated significantly between seasons and years, linking these changes to metal concentrations within coastal waters [52]. Mussels (*P. perna*) sampled from six South African harbours in 2008 had significantly higher metal concentrations than those from 2009, with these fluctuations credited to both variable metal discharges into harbours and large-scale changes in coastal upwelling [15]. A two-year study of marine contaminants within farmed mussels from Saldanha Bay found concentrations of both TMs and some POPs (pesticides and PCBs) fluctuated significantly within and between years, with certain metals and PCBs showing annual trends of accumulation and depletion [14,43]. These fluctuations can potentially be attributed to seasonal winter rainfall and wastewater transporting increased amounts of terrestrial pollutants into the ocean [53] or to the mussels’ reproductive cycle and gonadal tissue production, decreasing pollutant concentrations per specimen [48,54]. Within *C. meridionalis* from Cape Town, decreased metal concentrations in mussels were linked to raised water temperatures, causing elevated metabolic and excretion rates, which resulted in decreased exposure time within the gut and more TEs being egested [46].

Size: Since mussels only become sexually mature at a certain size, and gonadal production and spawning cause massive fluctuations in the flesh weight of adult mussels [39], significant differences in contamination levels have been noted between immature and mature specimens. Early studies on *M. edulis* found that Cd concentrations were significantly lower and more variable within sexually mature mussels than immature specimens, attributing this to biochemical processes that occur in the adult mussels’ sexual cycle [54]. *Mytilus galloprovincialis* mussels also showed decreasing TM concentrations with increasing dry flesh weight, although, in contradiction to Cossa et al. (1979), higher levels of inter-individual variability were detected in smaller specimens [47]. Research into human contaminant exposure through seafood focuses on the analysis of mussels of harvestable size (~7 cm), as these are the size consumers will be eating [55]. 

Depuration: Depuration involves submerging collected mussels in clean sea- or freshwater for approximately 24 h to allow the egestion of stomach contents and faeces prior to analysis; this ensures that analyses are not affected by physical or chemical contaminants that have not yet been assimilated into the mussels’ flesh [52,56,57,58]. Studies investigating human exposure to pollutants through seafood do not depurate mussels prior to analysis because aquaculture farms generally do not have the facilities for this, and mussels are therefore sold and eaten “as is” by consumers [59,60,61].

Location: The majority of studies on coastal marine pollution have found contamination levels within biota to increase with increasing proximity to large human settlements and industry [4,6,62]. However, this is not always the case with MP pollution; some of the most common plastics are buoyant and have long-term resistance to degradation, which allows them to be transported over long distances and often far from where they were introduced into the ocean [63,64]. 

Metallothioneins: Research has found that mussels have some ability to regulate the concentrations of certain TEs (like Cu, Zn, and Pb) within their flesh when they are bound to metallothionein proteins and then excreted [65,66,67]. This, at times, results in mussels being an unreliable indicator species for heavily polluted areas, as they possess the ability to down-regulate some metals’ accumulation under certain circumstances [66].

All the factors mentioned above can cause significant fluctuation in the concentrations of many commonly analysed pollutants and should be taken into consideration by researchers when investigating the contamination levels of mussels, whether for environmental monitoring or human exposure studies. 

#### 1.3.3. Targeted Mussel (or Bivalve) Species in South Africa

The southern African coastline varies greatly in temperature and environment, from the cold Benguela Current on the Western Coast to the warm Indian Ocean Current on the Eastern Coast, and therefore has many mussel species inhabiting the different niches available to them. The four most common mussel species are *Aulacomya ater* (the ribbed mussel), *Mytilus galloprovincialis* (the invasive Mediterranean blue mussel), *Choromytilus meridionalis* (the black mussel), and *Perna perna* (the brown mussel). *Aulacomya ater* is not often targeted by researchers because it tends to form dense beds below the intertidal mark [68], making collection difficult, and because this species is rarely exploited for human consumption. Together, the geographical distributions of the three selected mussel species cover the entire South African coastline and even extend into Namibia and Mozambique [41,42]. Additionally, *Mytilus galloprovincialis* is an extremely invasive mussel that originated in the Mediterranean and has spread internationally [69]; therefore, using this species in local research allows for easier comparison to similar studies from Europe and other countries. 

Three other bivalve species that are either farmed or wild collected by South African fishermen and seafood enthusiasts are the white mussel (*Donax serra*), the Cape rock oyster (*Striostrea margaritacea*), and the Pacific oyster (*Crassostrea gigas*) [20]. As a commercial aquaculture species, the Pacific oyster falls under the SAMSM&CP, and farmed samples are regularly tested for the same contaminants as commercial bivalves [70]. To date, only one study has reported on contaminants in Pacific oysters, finding that most TE concentrations were higher in farmed oysters than in farmed mussels from similar locations [71]. These data suggest that Pacific oysters may be more susceptible to the accumulation of marine contaminants and justify the need for both wild and farmed oysters to be targeted for future bivalve pollution research. The wild Cape rock oyster is not currently monitored for contaminants of any sort, potentially due to small standing stocks (when compared to ubiquitous mussels), the limited size of the fishery due to low levels of exploitation, and the cryptic nature of the species [20]. The white mussel is present at its highest densities along the South African West Coast, and the commercial fishery of this bait species is limited to seven locations within this zone [20]. This species has not been subjected to regular testing for marine contaminants, likely due to its popularity as a bait species (not consumed directly by humans). All three of the species mentioned above could be useful inclusions in a Mussel Watch Programme, as they would provide more detailed information on the status of contamination within coastal bivalves. *Donax serra*, in particular, could provide valuable data on pollutant contamination of sediments, as these mussels are burrowers and, therefore, may be exposed to different pollutants than bivalves living in the water column. 

### 1.4. Under-Monitored Marine Pollutants in South Africa

Mussels’ filter-feeding lifestyle exposes them to any physical or chemical contaminants present in the water column, which have been proven to both accumulate and concentrate within their flesh, posing a threat to the humans who consume them, as well as the entire marine food chain [28,72]. The South African Molluscan Shellfish Monitoring and Control Programme (SAMSM&CP) is run by the Directorate of Sustainable Aquaculture Management within the Chief Directorate of Aquaculture and Economic Development [70]. The aim of this programme is to guarantee the minimisation and management of any food safety risks associated with locally produced molluscan shellfish that are either sold locally or exported to international markets (with characteristically high standards). The targeted species for this programme include the Pacific oyster (*Crassostrea gigas*), Mediterranean mussel (*Mytilus galloprovincialis*), South African abalone (*Haliotis midae*), and local black mussel (*Choromytilus meridionalis*). The SAMSM&CP monitors the production phase of shellfish, testing produce for human health hazards such as contamination from microbiological organisms, bio-toxins, drug residues, dioxins, dyes, radionuclides, heavy metals, pesticides and polychlorinated biphenyls [70]. It is very important to note that this programme devotes most testing and analyses to short-term contamination events, which could severely damage human health [70]. These tend to be microbiological events such as *Escheria coli* or bio-toxin events such as harmful algal blooms (paralytic shellfish poisoning, diarrhetic shellfish poisoning). Microbiological contamination events require immediate action (e.g., closure of farms) to prevent human illness or even death from contaminated shellfish products and require high-intensity monitoring to determine strains, as well as how long shellfish farm closures need to continue. The programme also focuses exclusively on farmed shellfish and does not monitor levels of contamination on wild samples collected by recreational permit-holders, which puts wild collectors of coastal seafood at higher risk of eating shellfish that exceeds regulatory guidelines for human health. 

The contaminants that receive less monitoring are typically those that would not result in immediate human illness but rather create a long-term threat of contamination and accumulation within human tissue, such as toxic TEs, POPs (pesticides, polychlorinated biphenyls), and MPs. Long-term, repeated dietary exposure to toxic TE contaminants can result in an increased risk of cancer (arsenic, cadmium, lead), issues with neurological development in children (lead), impaired cerebral function in humans (mercury), etc. [73,74,75,76]. Similarly, human exposure to POP contamination in marine food chains can result in disruptions of the endocrine and immune systems, as well as cancers [77,78]. When compared to TEs and POPs, MP pollution research is relatively novel, and the potential negative human health impacts of MPs in the marine food chain are still being quantified. Recent work has identified MP contamination of food chains as a threat to human health because they may act as a vector for transporting and concentrating other contaminants within marine environments [79,80]. The following section elaborates on common sources of these pollutants in the marine environment and summarises available published research on contaminants in mussels from South Africa. 

#### 1.4.1. Trace Elements (TEs)

Miniscule amounts of TEs are present in all living organisms, with living tissues containing a wide range of trace metals [81]. There are three groups into which TEs are typically sorted; the first group includes essential TEs, which biological organisms require in order to function correctly but which can become toxic in excess [75]. For humans, these include copper (Cu), iron (Fe), manganese (Mn), molybdenum (Mo), selenium (Se), and zinc (Zn). The second group includes non-essential TEs, which currently have no known biological role in living organisms and typically have low toxicities. The third group includes the toxic TEs (including arsenic (As), cadmium (Cd), chromium (Cr), lead (Pb), mercury (Hg), and nickel (Ni)), which are harmful in even the smallest doses [35]. 

While natural sources of TEs exist within marine environments, most TE pollution in coastal zones has anthropogenic origins, such as urban run-off and stormwater, atmospheric emissions, industrial effluents, and large mining operations [82]. Elevated TE concentrations have been linked to river and stormwater inputs into the ocean, increasing human population densities and heavy industrial activities [4,83]. Marine food chains become contaminated with TEs through either direct contact with sediments or via their absorption into the phytoplankton, which filter-feeders sieve from the water column [76]. Trace element bioaccumulation in mussels is a useful tool for biomonitoring in marine ecosystems but is also a serious concern for the health of mussels and human seafood consumers [26,27,35]. 

#### 1.4.2. Persistent Organic Pollutants (POPs) 

Persistent organic pollutants (POPs) are complex and diverse chemicals produced by human industry, either intentionally or as unintentional by-products; natural processes cannot easily degrade POPs, and therefore they persist in the environment with concerning results [78]. The hydrophobic and lipophilic nature of POPs means they are bioavailable to plants and animals, increasing the chance of bioaccumulation in living creatures’ fat and bio-magnification up the food chain [34,84]. The negative side effects of these chemicals in animals (especially humans) are significant, including allergic reactions, disruption of nervous and immune systems, reproductive disorders and birth defects, cancer, and potentially death [78]. Persistent organic pollutants have been identified as an under-researched area of marine pollution research in South Africa, with this being attributed to the high cost of sample analysis [18].

The discovery that oceans and atmosphere facilitate the global transport of POPs [85] made it clear that the cooperation of all countries would be needed to control or eradicate these harmful substances. The United Nations Environmental Programme (UNEP) established the Stockholm Convention on persistent organic pollutants to either control or phase out a dozen priority POPs known to be extremely hazardous to human and ecosystem health [78]. South Africa entered the Stockholm Convention on POPs in September 2004 and has subsequently implemented various programs to identify, control, and safely dispose of hazardous POPs [86]. An exception was made for the use of DDT (dichlorodiphenyltrichloroethane) as malaria vector control in some South African Provinces, as it is considered essential for preventing human suffering [86]. As an agricultural country with a wide variety of crops, South Africa is also the largest importer of pesticides in sub-Saharan Africa [87], further justifying the need for POP pollution monitoring. Some major categories of POPs, as well as relevant South African studies using mussels as biomonitors, are discussed below. Well-known classes of POPs include organophosphate pesticides (OPPs), organochlorine pesticides (OCPs), polyaromatic hydrocarbons (PAHs), polychlorinated biphenyls (PCBs), and triazine herbicides; most of the pesticides within these classes have been well-researched, their toxicities and ability to accumulate in biota are well-known, and they have established regulatory limits for their detection in foodstuffs. 

Chemical contaminants of emerging concern: Contaminants of emerging concern (CECs) are novel chemical compounds that are produced by humans, have no established safe regulatory limits for their presence in human food sources, and are increasingly gaining scientific interest [88]. As human industries become more complex, the chemical wastes produced by them also increase in complexity and create identification obstacles for the scientists wishing to assess and quantify them. Therefore, there is a dire need for the evaluation of the quantities of these chemical contaminants within seafood to accurately assess their potential impacts on marine food chains and human health. 

#### 1.4.3. Microplastics (MPs)

Plastic is a synthetic, inexpensive, water- and corrosion-resistant material that is used in many industries worldwide and, due to its longevity, persists long after it has been discarded. Unfortunately, much of the world’s discarded plastic remains unrecycled and ends up in marine ecosystems, with studies estimating that 60–80% of marine litter is made up of plastics [89]. Microplastics (MPs) are pieces of plastic generally agreed by researchers to be <5 mm in length [90]. They are classified either as primary MPs, which enter the ocean as intentionally small particles (tiny, manufactured, plastic pieces such as microbeads from facial cleansers, nurdles, fibres from synthetic clothing), or from secondary MPs, which are a result of the fragmentation of larger marine plastics (e.g., plastic bags, netting, polystyrene) into smaller pieces through mechanical, chemical, and biological processes [91].

Studies have found MP pollution to be somewhat ubiquitous in the marine environment, with MP concentrations in sediments rising significantly with increasing human population densities [6,92]. Beach sediment samples from 18 sites spanning six continents (including Cape Town, South Africa) were all found to be contaminated with MPs, evidence of the extent of the issue [6]. The plastic types most typically encountered in the marine environment include polyvinylchloride, polyethylene, and polypropylene [91]. A recent meta-analysis of marine plastic polymer types found the most common plastics to be polyethylene (23%), polyester, polyamide and acrylic (20%), polypropylene (13%), and polystyrene (4%) [93]. 

The distribution of MP polymers varies between beach and bottom sediment samples, as well as between coastal and deep-sea samples, with this attributed to the diverse densities of plastics manufactured for different purposes [93,94]. Denser polymers (polyesters and acrylics) tend to sink faster into bottom sediments and deep-sea locations, while lower-density plastics (polypropylene and polyethylene) usually dominate in sea surface samples [93]. 

Microplastics are often further classified into five groups with varying origins: fragments, micro-pellets, fibres, films, and foams. Fragments are typically hard or flexible, jagged-edged pieces of secondary MP created through the breakdown of larger plastics and, as such, vary greatly in colour. In contrast, micro-pellets are primary MPs that are mostly hard, spherical, or cylindrical particles with a white or translucent colour [92,95]. Foams, or foamed polystyrene, are a white or yellowed colour, have a spongy texture, and are often clearly bio-fouled, while films consist of extremely thin, flat plastic sheets that are typically difficult to identify [92,95,96]. Lastly, fibres or filaments are thin, uniform plastic strands that commonly originate from the washing of synthetic textiles like polyethylene terephthalate (also known as polyester), polymethyl methacrylate (also known as acrylic), and polyamide (also known as nylon), which are all used extensively in modern clothing manufacturing [6,96]. Numerous studies on mussels, including one from Cape Town, South Africa, have found fibres to be the most frequently observed MP within samples [44,97,98,99,100,101]. 

Objectives: The first aim of this review is to assess the current state of marine pollution research within South African marine species to determine whether contaminant research is increasing or decreasing within the country and which anthropogenic marine pollutants are the least researched. The second aim of this review is to highlight how mussels have been used as pollutant biomonitors both worldwide and in South Africa, as well as the risk that mussels and other coastal seafood may pose to human consumers due to their exposure to marine anthropogenic contaminants. 

## 2. Materials and Methods

To summarise and assess the current state of research, a literature search was performed for published marine pollution articles and reviews in South Africa between June 2012 and December 2022, using the same terms included in the reviews by O’Donoghue and Marshall (2003) and Wepener and Degger (2012). For the year 2012, only papers published after March were included to avoid overlap with the previous literature search, which was published in July 2012 [18]. The terms included in the literature search were as stated by O’Donoghue and Marshall (2003), namely “South Africa, marine, pollution, estuarine, coast, nutrients, bacterial, microbial, sewage/sewerage, outfall, stormwater, chemical, sediment, bioaccumulation, biomonitoring, bioindication, ecotoxicology, organochlorine, organics, oil, petroleum, DDT, dieldrin, PCB, metal, zinc, mercury, cadmium, copper, lead, tin, radioactive, nuclear, thermal, litter”. Recorded details from each paper included the year of publication, the type of pollutant analysed, the publication journal, and which environmental compartment was analysed for contaminants. The databases used for the literature search were SCOPUS and Google Scholar. Only journal articles were included in the results (no theses, conference proceedings, or trade publications were included), and all research was performed using English search terms. This search resulted in 104 published papers on estuarine, coastal, and marine pollution research being identified between July 2012 and August 2022 (see Supplementary Material for a full list of included papers).

Publication bias: The majority of published papers assessed within this research are baseline studies reporting on detected contaminant concentrations within marine environments or biota. There is unlikely to be a “null” result for baseline studies, which would result in the authors choosing not to publish the data and, therefore, causing bias in the literature search results. However, there may be a publication bias due to “negative” results; severe contamination may be under-reported due to researchers fearing that publication could result in a future loss of access to funding or samples. Publication bias may also be present due to governmental research bodies being under no obligation to make their findings public, particularly if they reflect badly on their own capabilities. Government researchers often have access to more funding and specialised equipment for the analysis of samples, which could result in a publication bias detrimental to the field. 

## 3. Results

### 3.1. The Status of Marine Pollution Research in South Africa

The 104 collected papers were divided into three groups by topic: trace elements (TEs, also called “Metals”), persistent organic or inorganic pollutants (POPs), and microplastics (MPs) (Table 1). The most researched topics were TEs and persistent organic or inorganic pollutants, which were analysed in published work 42% and 38% of the time, respectively (44 and 40 analyses, respectively, Table 1), while MPs were only discussed 27% of the time (in 28 of the papers). The lower amount of research focus on MPs is likely because they are a relatively novel field of pollution research worldwide (particularly when compared to marine trace metal and POP research), and the number of published papers on MPs in South Africa showed a clear increase in frequency towards the end of the 2010s (Table 1). The most frequently studied environmental compartment was marine or estuarine biota, with the contamination levels of mussels, invertebrates, fish, birds, and other species being researched at least 65 times in the past decade (Table 1). The most frequently investigated category of marine contaminants in biota between 2012 and 2022 was fish (22% of total analyses), followed by mussels (18%) and invertebrates (12%). Marine pollution studies often included the analysis of water and sediment samples, with 25% and 27%, respectively, of the total research focusing on these environmental compartments (Table 1). 

### 3.2. Trace Elements in South African Mussels

Table 2 and Table 3 show that while there may be a perception that the South African marine environment is relatively pristine, certain toxic TEs such as Cr, Cd, Hg, and Pb have been detected in mussels at levels exceeding regulatory guidelines for seafood [102]. Concentrations of TEs were generally higher than those in less industrialised countries like Namibia while commonly being far lower than the detected levels within mussels from large, heavily industrialised countries like China (Table 2 and Table 3). The high variability in the reported concentrations of certain TEs within South African mussels (e.g., Cr, Fe, and Zn) makes comparison between different countries and areas more difficult.

### 3.3. Persistent Organic Pollutants in South African Mussels

The published research on mussels (summarised in Table 4) tentatively indicates that overall POP contamination levels within South African marine environments are moderate to low [32,43,103], particularly in comparison to more industrially developed areas like Spain, Hong Kong, and China [62,104,105,106], but far more widespread testing is necessary before decisive conclusions can be made on the health of the coastline as a whole. The only paper that performed a risk assessment of the detected concentrations of POPs determined one of the detected contaminants (a triazine herbicide called simazine) to pose a potential health risk to human consumers of wild seafood [107]. Within the period of 2012–2022, only one paper was published on chemical contaminants of emerging concern within mussels [108]. This paper found pharmaceutical products (e.g., diclofenac and acetaminophen) within mussels from Cape Town (South Africa) and is expanded on in the Discussion section below.

### 3.4. Microplastics in South African Mussels

In the 2012–2022 period, only two published local papers were available quantifying the microplastic load of South African mussels; one study quantified the MP load of wild mussels from Cape Town [44], while the other evaluated MPs in farmed mussels from aquaculture facilities in Saldanha Bay, South Africa [109]. 

**Table 2:** Common metal concentrations in bivalves from South African published research, with comparison values from the South African Department of Health shaded in grey (DW denotes values in dry weight; WW denotes values in wet weight).

Location	Species *	#	Unit	Cr	Mn	Fe	Co	Ni	Cu	Zn	As	Cd	Hg	Pb	Study Author/s
Cape Town	MG	Mn	µg/g DW		4.2	129.3			5.6	186.2		6.2	0.2	5.1	[52]
		Mx	µg/g DW		64.7	1309			43.9	1625.6		39.1	0.9	427.6	
Cape Town	MG	Min	µg/g DW		1.5	86.3			3.41	58.67		1.01		0.09	[110]
		Mx	µg/g DW		6.05	190.9			9.93	116.11		19.0		4.37	
Cape Town	MG	Mn	mg/kg DW	3.54	35.2		0.74	1.88			6.94	1.98	4.92	7.3	[111]
Cape Town	CM	Min	µg/g DW									0.6			[112]
		Mx	µg/g DW									16.3			
Richards Bay	PP	Mn	µg/g DW	0.62	8.9	151.1	0.33	0.6	1.9	16.1		0.14		0.3	[13]
		Mn	µg/g DW	60.6	64.7	420.2	142	20.5	111	229.7		3.9		4.1	
Saldanha	CM	Mn	mg/kg WW	0.1	1.6	11.3			1.2	15.5	1.8	0.4	0.003	0.2	[14]
		Mx	mg/kg WW	0.32	2.15	18.02			1.69	22.25	3.4	0.98	0.1	0.36	
	MG	Mn	mg/kg WW	0.2	0.5	25.1			0.7	25.9	1.8	1	0.004	0.5	[14]
		Mx	mg/kg WW	0.38	0.94	35.87			1.01	37.44	3.37	1.4	0.005	0.79	
Saldanha	MG	Mn	mg/kg DW	1.03	194	134	12.8			108	6				[71]
	CG	Mn	mg/kg DW	2.5	829	412	112			476	7.7				
SA coastline	MG	Min	µg/g WW	0.2	0.3	16	0.04	0.06		14	1.6				[16]
		Mx	µg/g WW	2.8	29.9	570	0.46	1.69		290	4.6				
SA West Coast	MG	Mn	mg/kg DW	2.4	3	150	0.64	3.1		200	4.4				[113]
SA South Coast	MG	Mn	mg/kg DW	2.4	7	250	0.26	1.6		180	7.2				
SA East Coast	MG	Mn	mg/kg DW	12.9	34	1980	4.68	15.1		90	9.4				
SA harbours	PP	Min	µg/g DW	0.6	1.4	3.4		0.03		2.8	0.08	0.03		0.09	[15]
		Mx	µg/g DW	19.4	168	665.4		2		618.9	32.1	6.8		12.9	
South African Max Limit Seafood			mg/kg WW	2					50	300	3	3	0.5	0.5	[102]

Note: * CM = *Choromytilus meridionalis*; CG = *Crassostrea gigas*; PP = *Perna perna*; PV = *Perna viridis*; MG = *Mytilus galloprovincialis*; ME = *Mytilus edulis*. # Mn = mean; Min = minimum; Mx = maximum.

**Table 3:** Common metal concentrations in bivalves from African countries and heavily industrialised countries (DW denotes values in dry weight; WW denotes values in wet weight).

Location	Species *	#	Unit	Cr	Mn	Fe	Co	Ni	Cu	Zn	As	Cd	Hg	Pb	Study Author/s
Namibia	CM	Mn	µg/g DW	0.75	1.1	2.6	0.68	1.09	1.07	2.2		0.88		1.95	[42]
	and PP	Mx	µg/g DW	1.2	1.98	3.32	1.21	1.95	1.4	2.92		1.55		2.38	
Morocco	MG	Min	µg/g DW					3.03	13.08	330.57				2.22	[114]
		Mx	µg/g DW					5.97	22.8	498.21				13.11	
Spain	MG	Min	mg/kg DW						3.89	141	6.39	0.357	0.037	0.57	[66]
		Mx	mg/kg DW						10.1	470	17.2	4.54	0.623	28.1	
Croatia	MG	Min	mg/kg DW	0.95					5.92	93		0.48	0.12	0.99	[4]
		Mx	mg/kg DW	6.15					369.31	563		1.72	10.3	14.79	
China	ME, MU,	Mn	µg/g DW	0.77				1.81	9.66	102.5		2.05		1.09	[115]
	and PV														

Note: * CM = *Choromytilus meridionalis*; CG = *Crassostrea gigas*; PP = *Perna perna*; PV = *Perna viridis*; MG = *Mytilus galloprovincialis*; ME = *Mytilus edulis*; MU = *Mytilus unguiculatus*. # Mn = mean; Min = minimum; Mx = maximum.

**Table 4:** Persistent organic pollutants in mussels from South African published research, with comparison values from studies in heavily industrialised areas shaded in grey.

Location	Bivalve Species *	TRZ **	OCPs **	PCBs **	PAHs **	Unit of Measurement	Study
SA harbours	PP			34–131	0.29–2.10	µg/g LW	[36]
Saldanha Bay	CM		83.8	6.9	25.4	ng/g DW (mean)	[43]
	MG		92.9	6.7	24.9	ng/g DW (mean)	[43]
Port Elizabeth	MG			14.48–21.377		ng/g WW	[103]
Cape Town	MG	157.8				ng/g DW	[107]
Spain	MG			0.67–39.2		µg/kg WW	[62]
Hong Kong	PV				27.76–74.17	µg/g LW	[106]
Hong Kong	PV			119–415		µg/g LW	[105]
China	PV and ME		14–640	1.0–13.0	456–3495	ng/g DW	[104]

Please note: * CM = *Choromytilus meridionalis*; PP = *Perna perna*; PV = *Perna viridis*; ME = *Mytilus edulis*; MG = *Mytilus galloprovincialis*; ** TRZ = triazine herbicide, OCPs = organochlorine phosphates, PCBs = polychlorinated biphenyls, PAHs = polyaromatic hydrocarbons.

## 4. Discussion

### 4.1. The Status of Marine Pollution Research in South Africa

The previous two reviews of local, published work (discussed in Section 1.2) indicated that South African marine pollution research was not following the international trend of increasing studies into the health and safety of coastal ecosystems for the protection of aquaculture operations, human recreational activities, and overall ecosystem health [17,18]. Potential reasons for this include the relatively pristine, undeveloped nature and image of the South African coastline (heavily industrialised countries have developed stricter pollution monitoring programs out of necessity), as well as the prohibitive cost of TE and organic contaminant analyses or private institutions lacking capacity or equipment necessary to analyse POP contaminants, particularly on a routine basis [17,18]. The recent literature search between 2012 and 2022, however, uncovered that publications on marine pollution research have more than doubled when compared to the previous time intervals of 1990–1999 and 2000–2012 [18]. Table 1 also shows an overall trend of an increasing number of marine pollution research studies from South Africa in the assessed period (2012–2022), peaking in 2020 at a total of 23 published papers. While this is a positive sign, it cannot be assumed that marine pollution research will continue this increasing trend in the country in the future, as the former boom of published research in the early 1980s was immediately followed by a drastic dip in publications [17], and therefore a longer time frame should be assessed before certain conclusions can be made.

The apparent recent increased interest in marine pollution research demonstrated in Table 1 could be due to several reasons, including (but not limited to) increasing concern over the state of South Africa’s water systems brought about by reports like the Green Drop on failing wastewater treatment works [11], statements and publications by pollutant researchers raising concerns over marine sewage outfalls [107,116], increasing awareness of the dearth of coastal pollution research by review articles [17,18,19], or an increase in overall public and private sector interest in the health of marine ecosystems due to awareness campaigns like those created around marine plastic litter in recent years [117]. The recent mass pollution of marine environments caused by flooding in Kwa-Zulu Natal (KZN, South Africa) in 2022 has also increased public awareness of the long-lasting effects of chemical and physical pollution. The floods resulted in extreme amounts of litter being washed away by rivers and deposited along the province’s beaches [118]. The long-term closure of numerous KZN beaches due to less visible pollution (from damaged industrial and sewage plants) has also raised major concerns for beachgoers, fisherman, and coastal foragers alike [119]. 

Despite the increase in the number of published works in the last 10 years, marine pollution research within South Africa still tends to cover small, disconnected sections of the coastline (often focused on aquaculture facilities or pollution hotspots like harbours), and therefore we cannot assume the condition of untested areas to be pristine [15,120]. A potential fix for this dearth of data could be the regular, coastal-wide collection and analysis of mussels for common pollutants (e.g., a South African Mussel Watch Programme). This would allow researchers to identify and protect truly pristine areas as well as identify pollution hotspots where interventions could be necessary and implemented before the ecological situation becomes so dire that it impacts the ocean’s economy of coastal wild capture fisheries and aquaculture. While a South African Mussel Watch already exists within the country, it rarely ever releases the data it collects to other researchers or publishes collected data [52]. The local Mussel Watch focuses on a select group of trace elements detected within mussel samples from Cape Town, South Africa [52]. This small area provides useful data but is not representative of the entire South African coastline. 

### 4.2. The Status of Under-Monitored Pollutants in South Africa

#### 4.2.1. Trace Elements

The concentrations of TEs in South African mussel samples (Table 2) are generally either similar to or lower than in heavily industrialised areas like Morocco [114], Spain [66], Croatia [4], or China [104]. Trace elements in South African mussels were higher than those found along the coastline of Namibia [42], a country just to the north of South Africa that has a significantly smaller population and lower industrialisation overall (Table 2 and Table 3). Table 2 also demonstrates the variation in which TEs are most analysed within South African research, with many studies not including either As, Hg, or Cr concentrations [13,46,52,71]; a likely reason for this is assumed to be either the high cost of analysis or a lack of the necessary analytical equipment. 

Most studies on TEs in mussels are centred around Cape Town and surrounds [46,52,111,112], which may be due to the dense population and resultant public concern over marine pollution and effluent disposal. The second most common research location was Saldanha Bay [14,43,45,71], which can be attributed to this being the location of all commercial mussel farming in South Africa [121]. While it is important and logical for research to focus on large cities and commercial aquaculture locations, the rest of the South African coastline could be considered under-monitored for TE contamination when compared to these focal areas. This lack of data along the general coastline could be harmful to wild mussel consumers. Wild mussels collected from the South African coastline in 2017 exceeded regulatory guidelines for Cr, Zn, As, and Se; the study recommended that human consumers should eat no more than 250 g per person per week of wild mussels harvested from around Cape Town and Durban due to potentially hazardous levels of Al, Cr, Co, Zn, As, and I [16]. Another study of mussels from South African harbours found maximum levels of Cr, As, and Pb, which exceeded local regulatory guidelines, noting the highest metal levels occurred in Cape Town, Mossel Bay, and Port Elizabeth harbours [15]. While these studies help raise awareness of pollution in South Africa, larger-scale projects covering the entire coastline over the long term are necessary to ascertain and monitor overall coastal health within the country to protect both the marine environment and human seafood consumers.

South Africa has regulatory guidelines that limit the amount of certain TEs within foodstuffs (Table 2) set by the Department of Health [102]. These maximum permissible limits (MPLs) are intended to ensure that consumers are not exposed to harmful levels of TEs, and while they are useful, recent research on human health risks associated with shellfish consumption now includes quotients and indexes such as Risk Quotients, Target Hazard Quotients and Total Hazard Indexes [122,123,124,125]. The United States Environmental Protection Agency (USEPA) first developed the Target Hazard Quotient (THQ) as an index value used to appreciate potential non-carcinogenic health risks associated with the consumption of a single toxic element [126]. If calculated THQ values for a given metal are >1, it indicates that lifelong consumption of the shellfish in question may result in severe negative health effects on human consumers [16,124]. The summary of all THQs for every tested trace element in a given sampling area is what determines the Total Hazard Index (HI), and a HI vale > 1 indicates that the cumulative consumption of all tested metals in shellfish poses potential health risks to consumers [16,124]. Risk Quotients (RQ) are calculated as a ratio between the estimated weekly intake (EWI) of shellfish in a country and provisional tolerable weekly intake (PTWI), with prescribed PTWI values representing acceptable weekly exposure levels to TEs in food while also taking into consideration that these may have a cumulative effect [16]. These index values can be used to help determine overall human TE exposure from shellfish consumption, as well as identifying intake limits (daily, weekly, monthly), which could reduce the risks to human shellfish consumers over a lifetime, and these indexes are not limited to the well-known “toxic” TEs such as Hg or Pb (all TEs can be toxic above a certain threshold, and it benefits humans to look at exposure cumulatively). Research on TE pollution in wild *M. galloprovincialis* mussels from Morocco recently found Cd and Cr to exceed local or international MPLs, and the additional information given by the calculated THQ and HI values indicated that while adults could consume moderate amounts of mussels from unpolluted areas without deleterious health effects, the risk for children was too high and therefore their consumption of mussels should be limited [124]. An Algerian study on the same species found a moderate human health risk from Fe and Cr and advised human consumers of wild mussels to eat no more than 500 g of mussel flesh per day [125]. To date, very little Southern African research has assessed the human health risk of consuming wild mussels via these indexes [16,113]. The two studies that were performed showed that recreational collectors should eat no more than 250 g of fresh mussels per week from South Africa (with even lower limits for Namibian and Mozambican mussels) to minimise their risk of negative health impacts from TEs [16,113]. More local, published research is needed to properly assess the benefits and risks of wild mussel consumption along the South African coastline.

Trace element concentrations above permissible limits mean that bivalve aquaculture operations like those in Saldanha Bay (which also functions as an industrial, deep-water harbour) need to regularly monitor contamination levels within mussels to ensure the health and safety of consumers and marine ecosystems [27,35]. When indigenous (*P. perna*) and artificial mussels (plastic devices containing metal-binding ligands) were transplanted into four large South African harbours [32], the live mussels in Saldanha Bay showed the lowest overall metal contamination of any site. The artificial mussels, however, had the highest overall metal contamination in Saldanha Bay; this could be due to water temperature differences between the study sites, causing differences in metabolic rates of the indigenous mussels but not their artificial counterparts [32]. A later study found mussels from aquaculture facilities in Saldanha Bay to be safe for human consumption overall but identified As, Cd, and Pb as potential contaminants of concern going forward [14]. Fortunately for mussel farmers in Saldanha Bay, the presence of both the SAMSM&CP and the Saldanha Bay Water Quality Forum Trust (who commission the yearly State of Saldanha Bay and Langebaan Lagoon Reports) ensure that the marine environment is well-monitored, allowing for the quick detection of novel pollutants and for rapid intervention to protect all industries of the Bay [121,127]. 

#### 4.2.2. Persistent Organic Pollutants

When compared to countries with larger human populations or higher levels of industrialisation (e.g., China, Hong Kong, and Spain), the contamination levels of OCPs, PCBs, and PAHs in South African mussels appear to be relatively low and are likely of little threat to the health of human consumers (Table 4). Research has consistently found that contamination levels of most pollutants increase in or near harbours and heavily populated areas [4]; since most of the POP research in South Africa occurred within harbours, these studies may represent the most POP-affected areas in South Africa. The conclusions of low contamination are difficult to substantiate, though, with so few published papers available on the topic. The wide variety of POPs in existence, the high cost of sample analyses, the limited availability of expensive equipment, and the complex internal standards necessary for POP analysis have all resulted in minimal research being conducted on these synthetic compounds in mussels collected from the South African marine environment [20].

With regard to chemical contaminants of emerging concern (CECs), there has been only one published paper that sought to identify and quantify novel marine pollutants within South African mussels [108]. This study found a wide range of pharmaceutical and personal care products within mussels from False Bay, a heavily populated, industrialised, semi-enclosed system in South Africa. While the concentrations of these contaminants were generally low, they may pose a risk to the ecology of the bay ecosystem, and it was also noted that the selected contaminants represent only a small amount of the wide range of contaminants that are likely present within False Bay due to human waste and sewage waters [108]. 

Research into the POP contamination of non-mussel species is increasingly prevalent within South Africa, with local studies investigating these contaminants in beached polyethylene pellets [128], chokka squid (*Loligo reynaudii*) [129], great white sharks (*Carcharodon carcharias*) [130], albacore tuna (*Thunnus alalunga*) [131], various dolphin species [132], waters and fish from multiple estuaries [133,134,135,136], the waters and sediments of Algoa Bay [137,138], and sediments from Richard’s Bay [139,140]. The results of the literature search showed that POP contamination research within South Africa is present but often limited in scope, occurring on only one species at a time or within a small sampling area. 

#### 4.2.3. Microplastics

Research on MPs in South African sea surface waters and seabirds was already present in the 1980s [141,142], and the body of local work has slowly grown since then. Published research on MPs has focused on quantifying these anthropogenic contaminants in seawater [143,144,145], in harbours [140,146], on sandy beaches [147,148], within polychaetes [149], and within varied fish species [150,151,152]. 

To date, there have only been two published papers on MPs in mussels from South Africa; the first found MPs to be present in 98% of wild mussel samples collected from around Cape Town, with 67% of the detected MPs being filaments or fibres [44]. The second study investigated retail mussels from supermarkets and wholesalers in Cape Town, finding that 87% of tested samples contained MPs and identifying a mean of 3.83 MPs per mussel, with 70% of the total detected MPs identified as filaments [109]. While the first study did not investigate plastic type, the second study found the major plastic polymer type to be polyethylene terephthalate (PET; 51.61%), with the second most common plastic identified as latex [109]. Both studies concluded that South African mussels showed MP contamination levels that were similar to or lower than international trends and called for further monitoring of these novel contaminants in the region [44,109]. 

## 5. Recommendations for Future Research

Regular testing for persistent marine contaminants would allow researchers and environmental protection agencies to potentially categorise and identify sources of pollution. The source of marine PAHs can be determined by whether they are predominantly high or low molecular weight [153], the source of pesticides can be determined by the type (e.g., pesticides used in households differ from those used in the farming industry), and different MP polymers can be attributed to different types of plastic pollution. A well-run, comprehensive, and transparent Mussel Watch programme along the South African coastline would therefore help identify imminent areas of concern, current pollution hotspots, and the sources of such pollution and create a baseline of environmental contamination levels to allow future research to determine whether overall contamination is increasing or decreasing over time. While the South African Department of Forestry, Fisheries and the Environment (DFFE) already has a Mussel Watch Programme, the data from this research are infrequently released to the public, if ever [52]. A potential solution for the current dearth of pollution data nationwide could be for the DFFE to coordinate with the private and public institutions that are already investigating this topic (Universities, water research groups, environmental impact assessment companies) within the country. Many South African universities have extensive research into marine pollution that may have spanned decades, e.g., plastic pollution research from the University of Cape Town (UCT) began in the 1980s [141,142], and researchers from the University of Johannesburg (UJ) reported on 34 years of metal monitoring within mussels from Richard Bay harbour [13]. There are also many scientists attempting to keep South African research into marine pollutants abreast with international trends, e.g., microplastics in local fish and seawater are being quantified by researchers at the Cape Peninsula University of Technology [44,109,154], as well as the University of Kwa-Zulu Natal [150], and both plastics [151] and novel marine pollutants are being identified by researchers at the University of the Western Cape [108,155]. There are already many private researchers working on quantifying existing and emerging marine pollutants within South Africa’s coastal ecosystems, but the research is often smaller-scale or covers one specific area or species, e.g., the private contaminant research that is funded by the Saldanha Bay Municipality (see Supplementary Material for more references). Larger studies spanning longer time periods and the whole coastline are necessary for a greater understanding of the status of marine contaminants in seafood and their potential impacts on human consumers. Coordination of research and funding opportunities from government departments such as the DFFE could result in larger, over-arching research projects that are executed by multiple research teams but have a common goal. The sharing of information, expertise, costs, and institutional facilities could be encouraged by the DFFE and would likely result in more comprehensive marine pollution research within the country. An example of the importance of involving multiple institutions within a contaminant analysis program can be seen in Table 2; two local studies analysed trace elements through Neutron Activation Analysis (NAA) and therefore did not determine concentrations of toxic Cd or Pb within the mussels [16,113], while another study was able to include these elements within mussel analyses due to the use of Inductively Coupled Plasma–Mass Spectrometry (ICP-MS) [14]. These researchers had different equipment available to them for trace element analysis and, therefore, covered different contaminants. Coordinated research projects may allow for more standardisation across the methodologies and data within published research, which creates information that is easier to compare over distance and time.

One of the largest issues currently facing South African researchers is a lack of available funding; the National Research Foundation has been subjected to continuous budget constraints [156], and concern has been raised over cuts to higher education funding and how this may impact the output of Universities and research scientists [157]. It has been suggested that South African researchers should look outside the country for project funding [158]; a large-scale Mussel Watch Programme with numerous stakeholders, which is coordinated by the DFFE, may have greater access to a variety of international research funds than numerous small, disjointed projects. 

The DFFE supervising a large-scale marine pollution monitoring project would also allow researchers from private institutions greater access to the governmental bodies that are required to address pertinent contamination findings. This will facilitate scientists advising actions for early pollution mitigation efforts such as the reform of waste-management practices (to ensure terrestrial pollution does not enter the marine environment through wind or dumping) or ineffective and outdated sewage systems, which have been identified as major sources of MP pollution to the marine environment [6]. South Africa has had issues with proper sewage treatment and management in the past [159], a trend that appears to be worsening in 2022 [11]. A large-scale, coordinated research project into the status of microplastic, trace elements, and persistent organic pollution within sewage and wastewater outputs would help identify current issues in the filtration of waste and potential remedies. 

More research is also needed into the effect of factors like species, sex, season, year, size, and location of sampled mussels with regard to pollution input sources because these differences can impact overall contaminant concentrations within mussel flesh and, where possible, should be controlled for when collecting samples. Long-term, repeated, annual analyses of varying mussel species and sizes from the same locations (as is typically performed within a Mussel Watch Programme) would assist researchers in determining spatial and temporal patterns of pollutant accumulation within mussels. 

Future research involving contamination levels of TEs in mussels from South Africa should strive to include risk assessments for human consumers (THQ, HI, RQ), as well as establishing weekly or monthly safe consumption limits of wild mussel consumers along the coastline. Risk assessments do not require extra analytical equipment or research facilities; they use equations, pre-determined factors such as consumer weight, and the determined contaminant concentrations within seafood products to determine the risk of selected seafood consumption. They, therefore, enhance the usefulness of already existing data on environmental contamination and allow it to be applicable to human consumers. Measures like this could help protect human consumers of wild mussels and other shellfish by identifying areas where mussel harvesting should be encouraged or limited due to the presence of one or more limiting trace element contaminants. 

The limited amount of research on the environmental effect of plastic pollution in Africa is a concerning issue for the continent, as reliable estimates of microplastic loads within ecosystems are necessary to assess the threat they pose to humans and the environment [160]. MP studies in South Africa are still uncommon, and to date, there are only two published papers on the MP loads of local mussels [44,109]; far more research is needed to truly assess the state of MP pollution along the coastline. One of the greatest issues plaguing MP research worldwide is that the novelty of the field has resulted in varying methodologies for the extraction of MPs from biological tissues, with older methodologies sometimes even causing the degradation, breakdown, or total loss of some MP particles [161,162]. It is, therefore, very important for South African scientists undertaking MP research in marine environments and species to ensure they are using the most up-to-date methodologies available to make the most of the limited research being conducted. Another issue within the field that requires more research is that laboratory experiments on MP ingestion in marine biota rarely use realistic contamination levels found within marine environments (experimental concentrations of MPs often exceed environmental concentrations by several orders of magnitude), and effort should be made to create accurate test situations [163].

## 6. Conclusions

Despite the perception that the South African marine environment remains pristine, the minimal amount of marine pollution research in the country over the past 30 years makes this assumption hard to prove. An increasing number of marine pollution studies in the country between 2012 and 2022 indicate concerns over this long-held perception. The growing aquaculture industry (specifically non-fed mariculture of oysters and mussels) creates both food security and much-needed jobs in South Africa, and effort should, therefore, be made to protect the marine coastal environments essential for aquaculture operations from excessive or uncontrolled terrestrial pollution. An advantage for local pollution researchers is the widespread presence of the invasive, farmed mussel *M. galloprovincialis*; this international species is often used as a biomonitor in marine pollution research in Europe, which allows for some comparison of contaminant loads between more developed countries and South Africa. A transparent, coastal pollution monitoring programme like the Worldwide Mussel Watch, which regularly collects mussel biomonitors and analyses them for persistent marine contaminants such as trace elements, organic pollutants, and microplastics, would allow marine scientists to identify hotspot areas of emerging or current pollution and potentially implement mitigation measures to control or minimise point-source inputs of terrestrial pollution (e.g., better waste and sewage management practices). A potential approach to a beneficial local Mussel Watch Programme could be for the South African Department of Forestry, Fisheries, and the Environment to coordinate and fund research efforts through private and public interested and affected parties (e.g., University researchers and aquaculture facilities). This research could also be used to determine area-specific coastal consumption rate limits for wild mussel consumers to minimise the risks associated with wild shellfish consumption. The documented decline in overall marine pollution research within South Africa (though currently shown to be increasing between 2012 and 2022) and the limited research in other African countries is a concern for all ocean users and desperately needs to be addressed by large-scale research projects covering the region as a whole.

## Figures and Tables

**Table 1:** A summary of the research topics covered according to pollutant type and environmental compartment analysed in papers published on marine pollution research in South Africa between 2012 and August 2022.

		Pollutant Type			Environmental Compartment Analysed							
Year	Publications/Year	Metals	* MPs	* POPs	Review	Water	Sediment	Mussels	Invertebrates	Fish	Birds	Other
2012	3	2	0	1	0	1	0	2	1	0	0	0
2013	2	2	1	2	2	0	0	0	0	0	0	0
2014	7	6	1	0	0	1	2	2	0	0	1	Micro-organisms (1)
2015	7	3	2	3	0	2	2	2	2	2	1	0
2016	13	7	2	4	0	1	2	1	0	7	0	Turtles (1); Dolphins (1)
2017	10	4	2	5	0	5	5	0	0	4	0	Micro-organisms (1)
2018	3	1	0	2	0	0	1	1	0	0	0	Corals and sponges (1)
2019	14	2	3	9	0	6	3	2	1	2	1	0
2020	23	8	12	5	7	5	5	4	4	5	0	Corals (1)
2021	17	7	5	6	2	4	6	3	2	3	0	Algae (1)
2022	5	2	0	3	0	1	2	2	2	0	0	Algae (1)
Total	104	44	28	40	11	26	28	19	12	23	3	8

Please note: Total numbers for pollutants and environmental compartments analysed refer to the number of times each aspect was investigated or mentioned and may exceed the total number of papers tallied due to a significant number of papers covering multiple pollutant types and/or a variety of species, trophic levels, etc. * MPs = microplastics, POPs = persistent organic pollutants.

## Data Availability

All data are contained within the article and are available in Supplementary Material.

## Supplementary Materials

The following supporting information can be downloaded at: https://www.mdpi.com/article/10.3390/foods12213983/s1.
